# Supplementary material for: Preoperative Predictors of Recurrent Tricuspid Regurgitation After Annuloplasty: Insights into the Role of 3D Echocardiography
Source: Diagnostics (Basel). 2024 Nov 10;14(22):2515. doi: 10.3390/diagnostics14222515 (PMC11592411; doi:10.3390/diagnostics14222515)
Supplement: Supplementary file 1 [file diagnostics-14-02515-s001.zip › diagnostics-3256734-supplementary.pdf]

Supplementary Tables

Table S1. Inclusion and exclusion criteria.

**Inclusion criteria**
Adult subjects who are ≥ 18 years old.
Patients with moderate-severe functional tricuspid regurgitation associated with left heart valve disease undergoing concomitant tricuspid annuloplasty during left heart surgery.<br>Left heart surgical procedures include:<br>a. Mitral valve repair,<br>b. Mitral valve replacement,<br>c. Aortic valve replacement,<br>d. Mitral valve repair combined with aortic valve replacement,<br>e. Mitral valve replacement combined with aortic valve replacement
Patients must be hemodynamically stable, without requiring inotropic support or mechanical circulatory support.
Patients must have been on optimized and stable guideline-directed medical therapy for heart failure or other cardiac conditions.
Patients must have a life expectancy of at least 12 months, not limited by non-cardiac comorbidities such as advanced cancer or other terminal illnesses.
Must sign an informed consent form (ICF) indicating that he or she understands the purpose of, and the procedures required for, the study. Subjects must be willing and able to adhere to the prohibitions and restrictions specified to study protocol.
**Exclusion criteria**
Patients with primary tricuspid regurgitation.
Other pathologies which may influence the results:<br>a. congenital heart disease or other causes of precapillary pulmonary hypertension,<br>b. chronic pulmonary disease.
Patients with significant ischemic heart disease (assessed by coronary angiography).
Patients with active or recent infective endocarditis within the past 6 months.
Patients with end-stage renal disease or severe renal dysfunction (e.g., eGFR <30 mL/min or on dialysis).
Any other ongoing infections (e.g., sepsis) that may impair recovery or increase surgical risks.
Patients who have undergone prior tricuspid valve repair or replacement.
Patients with an implanted transvalvular permanent pacemaker.
Patients with poor quality 2D echocardiography images that were not suitable for 3D echocardiography.

Patients with severe cognitive impairment, such as advanced dementia or with uncontrolled psychiatric illnesses (e.g., schizophrenia, bipolar disorder, who are unable to provide informed consent or follow postoperative care plans.

Table S2. Clinical and echocardiographic characteristics of study population.

	Effective TAP group *n*=50 (75.8 %)	Recurrent TR group *n*=16 (24.2 %)	*p*-value
**Clinical characteristics**			
Age, years	67 (14)	73 (14)	0.059
Male, %	58	50	0.578
Body mass index, kg/m^2	26.6 (6.7)	29.0 (10.6)	0.224
**LV parameters**			
End-diastolic diameter index, mm^2	27.5 (5.6)	28.3 (5.3)	0.616
End-diastolic volume index, ml/m^2	70.7 (37.4)	58.9 (28.2)	0.109
End-systolic volume index, ml/m^2	35.5 (30.2)	30.6 (23.1)	0.302
LV EF, %	52.4 (18.8)	50.4 (17.5)	0.811
**LA parameters**			
Diameter, mm	48 (6.3)	53 (12.8)	0.135
Volume index, ml/m^2	57.1 (35.4)	72.1 (33.2)	0.120
**PA parameters**			
Max. systolic PAP, mmHg	53 (22)	56 (17)	0.736
Mean PAP, mmHg	38 (13)	39 (12)	0.319

LV – left ventricle, EF – ejection fraction, LA – left atrium, PA – pulmonary artery, PAP – pulmonary artery pressure.

Table S3. Relation of conventional echocardiographic parameters with recurrent TR.

	Univariate			Model I		
	OR	95% CI	*p*-value	OR	95% CI	*p*-value
**Left-heart side parameters**						
LV end-diastolic diameter index, mm^2	1.02	0.89-1.18	0.773	1.10	0.90-1.35	0.336
LV end-diastolic volume index, ml/m^2	0.98	0.95-1.01	0.098	0.98	0.95-1.01	0.124
LV EF, %	1	0.96-1.05	0.954	0.99	0.94-1.04	0.715
LA volume index, ml/m^2	1.02	1-1.04	0.074	1.02	1.01-1.04	**0.04**
**Right-heart side parameters**						
RV parasternal diastolic diameter, mm	1.22	1.06-1.40	**0.006**	1.25	1.07-1.46	0.508
RV basal diameter, mm	1.18	1.06-1.31	**0.002**	1.19	1.06-1.32	**0.002**
RV basal diameter index, mm/m^2	1.34	1.09-1.65	**0.005**	1.3	1.05-1.6	**0.017**
RV middle diameter, mm	1.09	1.01-1.19	**0.033**	1.1	1.01-1.12	**0.031**
RV middle diameter index, mm/m^2	1.19	1.03-1.38	**0.02**	1.17	1.01-1.36	**0.049**
RV sphericity index, %	1.07	1.01-1.13	**0.02**	1.06	1.01-1.12	**0.048**
RV end-diastolic area, cm^2	1.08	0.99-1.17	0.061	1.12	1.02-1.23	**0.019**
RV end-diastolic area index, cm^2/m^2	1.21	1.02-1.44	**0.033**	1.24	1.35-1.50	**0.024**
RV end-systolic area, cm^2	1.1	0.99-1.21	0.057	1.17	1.03-1.32	**0.013**
RV end-systolic area index, cm^2/m^2	1.26	1.02-1.56	**0.034**	1.35	1.06-1.72	**0.015**
FAC, %	0.96	0.88-1.04	0.299	0.94	0.86-1.03	0.184
TAPSE, mm	0.92	0.81-1.05	0.222	0.91	0.79-1.04	0.168
RV S', cm/s	0.96	0.78-1.12	0.672	0.92	0.74-1.15	0.455
RV end-diastolic volume, ml	1.01	0.99-1.02	0.128	1.02	0.99-1.03	0.06
RV end-systolic volume, ml	1.01	0.98-1.03	0.1	1.02	1-1.05	**0.047**
RV stroke volume, ml	1.02	0.99-1.05	0.27	1.03	0.99-1.07	0.16
RV EF, %	0.96	0.86-1.06	0.423	0.94	0.83-1.06	0.281
RV septal wall strain, %	1.07	0.90-1.27	0.476	1.11	0.91-1.34	0.310
RV lateral wall strain, %	1.02	0.87-1.19	0.855	1.11	0.99-1.24	0.073
RA diameter, mm	1.13	1.03-1.23	**0.007**	1.19	1.06-1.33	**0.003**
RA length, mm	1.11	1.03-1.20	**0.01**	1.16	1.05-1.28	**0.004**
RA area, cm^2	1.12	1.03-1.23	**0.01**	1.16	1.04-1.29	**0.006**

RA volume, ml	1.02	1.01-1.03	**0.015**	1.02	1-1.04	**0.013**

LV – left ventricle, EF – ejection fraction, LA – left atrium, RV – right ventricle, FAC – fractional area change, TAPSE – tricuspid annular plane systolic excursion, S' – tricuspid lateral annular systolic velocity, RA – right atrium.

Table S4. Prediction of recurrent TR.

Variables	AUC	*p*-value
**Left-heart side parameters**		
LV end-diastolic diameter index, mm^2	0.542	0.611
LV end-diastolic volume index, ml/m^2	0.366	0.109
LV EF, %	0.480	0.811
LA volume index, ml/m^2	0.630	0.120
**Right-heart side parameters**		
RV parasternal diastolic diameter, mm	0.747	**0.003**
RV parasternal systolic diameter, mm	0.744	**0.004**
RV basal diameter, mm	0.763	**0.002**
RV basal diameter index, mm/m^2	0.737	**0.006**
RV middle diameter, mm	0.709	**0.012**
RV middle diameter index, mm/m^2	0.685	**0.027**
RV sphericity index, %	0.325	**0.041**
RV end-diastolic area, cm^2	0.667	**0.046**
RV end-diastolic area index, cm^2/m^2	0.699	**0.017**
RV end-systolic area, cm^2	0.653	0.067
RV end-systolic area index, cm^2/m^2	0.699	**0.017**
FAC, %	0.600	0.231
TAPSE, mm	0.579	0.347
RV S', cm/s	0.538	0.648
RV end-diastolic volume, ml	0.634	0.211
RV EF, %	0.637	0.202
RV lateral wall strain, %	0.546	0.668
RA diameter, mm	0.735	**0.005**
RA length, mm	0.746	**0.003**
RA area, cm^2	0.723	**0.008**
RA volume, ml	0.688	**0.026**

LV – left ventricle, EF – ejection fraction, LA – left atrium, RV – right ventricle, FAC – fractional area change, TAPSE – tricuspid annular plane systolic excursion, S' – tricuspid lateral annular systolic velocity, RA – right atrium.
